# The validity and reliability of the Turkish version of the Body Esteem Scale for Adolescents and Adults (BESAA) for children

**DOI:** 10.3906/sag-1902-171

**Published:** 2020-04-09

**Authors:** Umut Ece ARSLAN, Lütfiye Hilal ÖZCEBE, Hande KONŞUK ÜNLÜ, Sarp ÜNER, Mahmut Saadi YARDIM, Özgür ARAZ, Terry T-K HUANG

**Affiliations:** 1 Department of Health Research, Institute of Public Health, Hacettepe University, Ankara Turkey; 2 Department of Public Health, Faculty of Medicine, Hacettepe University, Ankara Turkey; 3 College of Business, University of Nebraska, Lincoln, NE USA; 4 Graduate School of Public Health and Health Policy, City University of New York, New York, NY USA

**Keywords:** Body esteem, children, survey, scale

## Abstract

**Background/aim:**

Body esteem (BE) is defined as the self-evaluation of one’s own body or appearance. The Body Esteem Scale for Adolescents and Adults (BESAA) consists of three subscales: BE-appearance, BE-weight, and BE-attribution. Though initially developed for adolescents and adults, the use of the scale has recently increased in health-related research on children. This study aimed to assess the validity and reliability of the Turkish version of the BESAA for children.

**Materials and methods:**

The participants in the study were 4th grade children (aged 9–10 years) in Ankara, Turkey. The validity of the scale was evaluated through exploratory and confirmatory factor analyses. Internal consistency and test-retest reliability were assessed using Cronbach’s alpha and Spearman’s rho correlation coefficient, respectively.

**Results:**

The Turkish version of the BESAA for children includes BE-weight, BE-appearance, and BE-attribution subscales. The scale demonstrated good model fit statistics (chi-square/df = 3.41, P < 0.001) and good internal consistency for BE-weight (α = 0.85), BE-appearance (α = 0.76), and BE-attribution (α = 0.69). According to our findings, test-retest reliability of the three subscales was in the moderate/acceptable range for children (r = 0.57–0.68, P < 0.01).

**Conclusion:**

The Turkish version of the BESAA can be used to measure BE in terms of appearance, weight, and attribution in children.

## 1. Introduction

Body esteem (BE) is defined as the “self-evaluation and self-esteem of one’s physical appearance” [1]. According to Mendelson et al. [1], BE comprises three dimensions: BE-appearance (“feelings about one’s general appearance”), BE-weight (“feelings about one’s weight”), and BE-attribution (“evaluations attributed to others about one’s body and appearance”). The media, social environment, and perception of family affect the BE of all children, even those of normal weight. Studies, especially those conducted in the last few decades, have pointed out the importance of BE, body image, and body dissatisfaction among children and adolescents [2].

Children with poor BE consider themselves as ugly, sloppy, lazy, stupid, unhappy, less competent, isolated, and lacking in self-discipline, motivation, and self-control [3]. Poor BE in children may make them prone to risky behaviors such as substance use [4] and has been confirmed as a risk factor for eating disorders or dysregulated restrained eating [5]*. *Obesity, in turn, causes stigmatization, which can reinforce poor BE. Children who are obese experience social rejection and discrimination in their social environment, and depression is one of the potential consequences of obesity. Therefore, BE is one of the most investigated topics related to children with eating disorders and obesity [6].

There is some discussion in the literature on the reliability and validity of the BE Scale for Adolescents and Adults (BESAA). Cragun et al. [7]. examined the reliability and validity of the BESAA for a population of early adolescent males and females. Data were collected from children in one middle school in Florida (n = 299) with a mean age of 11.9 years (SD = 0.54, range = 11–13, male: 48.8%). Only the BE-appearance and BE-weight subscales were employed, not the BE-attribution subscale. They reported that the items “My looks will help me get dates” and “My looks will help me get a job” were not included in the BE-attribution subscale. The third item from the BE-attribution subscale of the BESAA (“I’m as nice looking as most people”) was included as part of the BE-appearance subscale. The two remaining items of the BE-attribution scale of the BESAA were not included in the subscale because these were insufficient to create a separate subscale. The authors found that the subscales exhibited good internal consistency reliability, with Cronbach’s alphas ranging from 0.90 to 0.93 for male and female children. Confalonieri et al. [8] investigated the validity and reliability of the BESAA using an Italian sample comprising 674 Italian adolescents aged 11–16 years (M = 13.33, SD = 2.1). They confirmed good reliability and internal validity of the 14-item Italian version of the BESAA, which comprised three subscales for adolescents. Mak et al. [9] investigated the internal consistency of the BESAA by using Cronbach’s alpha coefficient. They also examined the associations of BE with gender, age, and body mass index (BMI) among 905 adolescents in Hong Kong (M = 14.7, SD = 1.9) using the BESAA scale. The reliability coefficients of appearance, attribution, weight and overall were 0.53, 0.85, 0.74, and 0.78, respectively. Rousseau et al. [10] validated the French version of the BESAA using a sample of 835 adolescent girls and young adults (M = 16.62, SD = 1.50). Their exploratory factor analysis showed that three factors, namely weight, appearance, and desire, modify the negative effects associated with general appearance. Furthermore, the goodness-of-fit of the three-factor model was satisfactory.

The BESAA, the most widely used scale in this regard developed by Mendelson et al., was first introduced in 1996 [11]. In 2011, it was developed into a self-reported questionnaire for adolescents and adults [1]. This study aimed to examine the validity and reliability of the Turkish version of the BESAA for children. 

## 2. Materials and methods 

### 2.1. Study design and participants

A cross-sectional descriptive study was employed. This study was part of the Child Obesity Study of Ankara (COSA), which aimed to investigate the prevalence of obesity and related factors in Turkey. The COSA was conducted by Hacettepe University Institute of Public Health in Ankara, Turkey, and the University of Nebraska Medical Center in the United States of America. Further details of the study can be found in Haley et al. [12] and Steenson et al. [13]. The sample for the study was 2066 school children (aged 9–11 years). The COSA was a population-representative survey of children in the 4th grade in 46 schools in Ankara, Turkey, and their parents, conducted during the 2014–2015 school year. Ankara was selected for the purposes of the study by ranking counties according to socioeconomic status (SES) level (low, middle, high), based on previously reported socioeconomic indicators and social structures [14]. In total, 46 schools (15 schools from a low SES county, 17 from a medium SES county, and 14 from a high SES county) were included in the study. The schools were selected from each SES stratum by using probability proportional-to-size (PPS) methodology. 

In this study, the number of children in the validity analysis was based on the full sample of children in the COSA (n = 2066): with 1100 (53.2%) with low SES, 715 (34.6%) with medium SES, and 251 (12.2%) with high SES. Test-retest reliability was investigated in a separate substudy using a similar SES-stratified random sampling method. Two primary schools in each stratum were randomly selected, and 641 children were enrolled in the study [low SES: n = 243 (38.0%), middle SES: n = 205 (32.0%), high SES: n = 193 (30.0%)]. The same questionnaire was administered to these children twice 3 weeks apart to examine the test-retest reliability of the BESAA. 

### 2.2. Measurement

The BESAA consists of 23 items (10 negative and 13 positive items). The responses are indicated on a five-point Likert scale, ranging from 0 (“never”) to 4 (“always”) (Table 1). The scale has negative items (4, 7, 9, 11, 13, 17, 18, 19, and 21), which are reverse-scored. The 23 items of the BESAA consist of three subscales: BE-appearance (general feelings about appearance; items 1, 6, 7, 9, 11, 13, 15, 17, 21, and 23, Cronbach’s alpha = 0.92; accounted for 49.3% of the variance), BE-attribution (evaluations attributed to others about one’s body and appearance; items 2, 5, 12, 14, and 20, Cronbach’s alpha = 0.81; accounted for 10.4% of the variance), and BE-weight (weight satisfaction; items 3, 4, 8, 10, 16, 18, 19, and 22, Cronbach’s alpha = 0.94; accounted for 5.9% of the variance) [1]. Mendelson et al.’s [1] study sample included 1334 participants (763 girls and women, 571 boys and men) drawn from English-speaking elementary schools, high schools, universities, and a junior college in Montreal, Quebec, Canada, aged between 12 and 25 years (median = 16.8 years). The retest sample of the study comprised 97 junior college students (61 women and 36 men) examined three months after their initial test. The test-retest correlations were high (BE-appearance: r = 0.89, P < 0.001, BE-weight: r = 0.92, P < 0.001, and BE-attribution: r = 0.83, P < 0.001). Higher scores on the three subscales indicated more positive BE [1]. 

**Table 1:** Factor loadings (>0.40), Cronbach’s alpha, and test-retest reliability for the Body Esteem Scale for Adolescents and Adults introduced by Mendelson et al. [11].

Number of item	Items	Appearance	Attribution	Weight
11	I wish I looked like someone else	0.86		
7	There are lots of things I’d change about my looks if I could	0.80		
9	I wish I looked better	0.77		
13	My looks upset me	0.76		
17	I feel ashamed of how I look	0.71		
21	I worry about the way I look	0.69		
6	I like what I see when I look in the mirror	0.51		
23	I look as nice as I’d like to	0.50		
15	I’m pretty happy about the way I look	0.50		
1	I like what I look like in pictures	0.42		
2	Other people consider me good looking		0.83	
20	My looks help me to get dates		0.77	
12	People my own age like my looks		0.74	
5	I think my appearance would help me get a job		0.64	
14	I’m as nice looking as most people		0.61	
8	I’m satisfied with my weight			0.96
10	I really like what I weigh			0.92
16	I feel I weight the right amount for my height			0.89
19	My weight makes me unhappy			0.77
4	I’m preoccupied with trying to change my body weight			0.73
18	Weighing myself depresses me			0.69
22	I think I have a good body			0.61
3	I’m proud of my body			0.58
	Explained variance (%)	49.3	10.4	5.9
	Cronbach’s alpha	0.92	0.81	0.94
	Test-retest reliability	0.89	0.83	0.92

### 2.3. Translation and cross-cultural adaptation for the Turkish version of the BESAA for children 

The BESAA was translated into Turkish by the Turkish research team and then back-translated into English by a professional translation company in the United States. The Turkish and American research teams evaluated the original and English-translated versions of the scale. If there were no differences between the two English versions of the scale, the Turkish team further reviewed the Turkish translation. The Turkish scale was piloted among 20 children from a school not included in the survey sample. The scale was administered to the children, who were asked whether there were any issues with the scale translation or adaptation. After the research team reviewed the scale, it was examined by five Turkish linguists working at the primary school level who were members of the Education Faculty of Hacettepe University. The provincial directorate of the Ministry of National Education reviewed the scale to grant permission to conduct the study in the selected schools. The Ankara Provincial Directorate did not approve one item (“My looks help me to get dates”); thus, this item was excluded from the scale.

### 2.4. Statistical analysis

Statistical analyses were performed using SPSS and AMOS 23.0 (IBM Corp., Armonk, NY, USA). Scale validity was evaluated using exploratory factor analysis (EFA) and confirmatory factor analysis (CFA). The EFA was performed using the principal component analysis with oblimin rotation (Kaiser normalization) for the factor structure. The Kaiser–Meyer–Olkin statistic and Bartlett’s test of sphericity were carried out to check for sampling suitability and factor structure. If an item loaded (<0.40) on more than one factor, it was removed from the scale [15]. Internal consistency was assessed using Cronbach’s alpha. The CFA was employed after the EFA to determine the goodness of fit of the three-factor model of the BESAA after EFA. The following parameters were used to evaluate model fit: the chi-square to df ratio (CMIN/df), root mean square error of approximation (RMSEA), standardized root mean square residual (SRMR), goodness of fit index (GFI), adjusted goodness of fit index (AGFI), comparative fit index (CFI), and Tucker–Lewis Index (TLI). The following criteria were used to assess model fit: CMIN/df < 5, RMSEA < 0.08, SRMR < 0.05, GFI > 0.90, AGFI > 0.90, CFI > 0.95, and TLI > 0.95 [16,17]. Cronbach’s alpha was used to evaluate the internal consistency of each subscale (BE-appearance, BE-weight, and BE-attribution) and the overall scale, and Spearman’s correlation coefficient was used to assess test-retest reliability. The Mann–Whitney U test was employed for comparison of groups by gender. P < 0.05 was considered to be statistically significant.

Approval from the Provincial Directorate of the Ministry of National Education was obtained to conduct the study in the selected schools. In addition, ethical approval was obtained from the Noninterventional Clinical Research Ethics Board at Hacettepe University, Ankara, Turkey (GO 14/429-07). Each school in the research sent information the concerning the study to parents. The consent of parents and children was obtained before the data collection.

## 3. Results

The original version of the BESAA comprises 23 items. The Turkish version of the BESAA applied to the children in our study consists of 22 items, because, as mentioned, one item was not approved by the provincial directorate of the Ministry of National Education. In total, 1648 children (47.3% boys and 52.7% girls), aged 9–11 years (median = 10.0), completed the BE Scale, and their data were used to analyze the validity of the BE Scale for children.

### 3.1. Validity study

A factor analysis was performed to assess the construct validity of the BESAA. Several items were dropped from the scale because they loaded on different dimensions. These were item 15 [“I am pretty happy about the way I look” (BE-appearance)], 23 [“I look as nice as I’d like to” (BE-appearance)], and 6 [“I like what I see when I look in the mirror” (BE-appearance)]. Items 18 [“Weighing myself depresses me” (BE-weight)] and 19 [My weight makes me unhappy” (BE-weight)] loaded on appearance and item 1 [“I like what I look like in pictures” (BE-appearance)] loaded on BE-attribution. Item 4 [“I am preoccupied with trying to change my body weight” (BE-weight)] did not significantly (i.e. not ≥0.40) load on any of the components. After these items were dropped, the results of the EFA for the BESAA were recalculated. Table 2 presents the 3-factor solution (BE-appearance, BE-weight, and BE-attribution), and together, these factors explained 58.981% of the total variance.

**Table 2:** Results of the explanatory factor analysis of the Turkish version of the Body Esteem Scale for Adolescents and Adults for children (n = 1648).

	Factor loadings		
Items of Turkish version	Weight	Appearance	Attribution
10. Kilomdan gerçekten memnunum	0.906		
8. Kilomdan memnunum	0.878		
16. Boyuma göre doğru kiloda olduğumu hissediyorum	0.697		
22. Güzel bir vücudum olduğunu düşünüyorum	0.550		
3. Vücudumla gurur duyuyorum	0.468		
17. Görünüşümden utanıyorum		0.847	
13. Görünüşüm beni üzüyor		0.842	
21. Görünüşüm beni endişelendiriyor		0.833	
11. Başka birine benzemek isterdim		0.737	
7. Yapabilecek olsaydım, görünüşümde değiştirmek istediğim çok şey var		0.543	
9. Daha iyi görünmek isterdim		0.443	
2. Diğer insanlar benim iyi göründüğümü düşünürler			0.752
12. Yaşıtlarım görünüşümü beğenirler			0.606
5. Görünüşümün, iş bulmamda yardımcı olacağını düşünüyorum			0.552
14. Pek çok insan kadar hoş görünüyorum			0.517
Initial eigenvalue	5.066	2.759	1.022
Explained of variance (%)	33.774	18.396	6.811
Explained of cumulative variance (%)	33.774	52.169	58.981

The CFA was conducted for the 15-item, three-factor model (Figure). The model demonstrated good model fit statistics (chi-square/df = 3.406, P < 0.001) and the goodness of fit values for the confirmatory model were acceptable: RMSEA = 0.039, SRMR = 0.040, GFI = 0.979, AGFI = 0.967, CFI= 0.981, and TLI = 0.975. 

**Figure:**
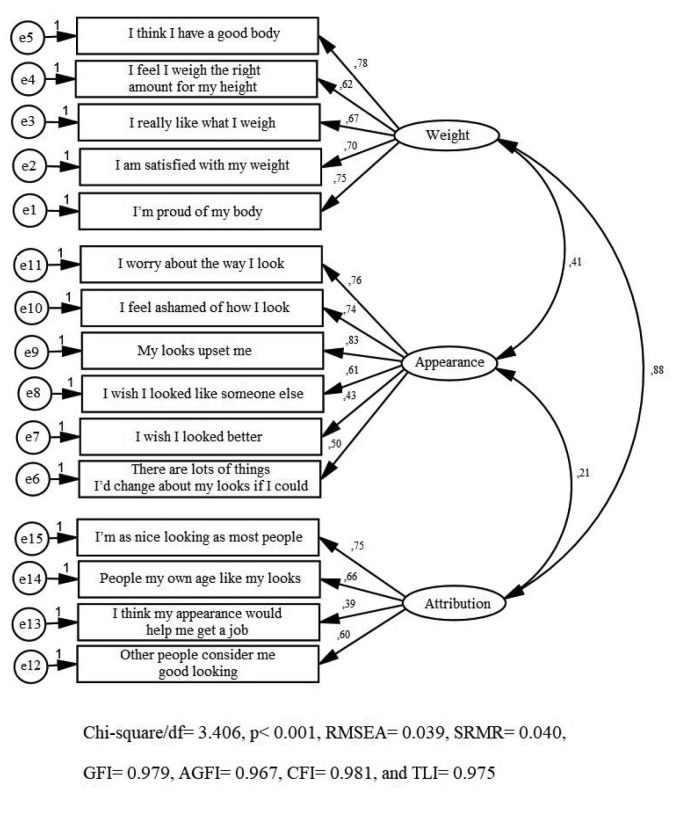
Confirmatory factor analysis and model fit statistics of the Turkish version of the Body Esteem Scale for Adolescents and Adults for children.

### 3.2. Test-retest reliability and internal consistency 

The final Turkish version of the BESAA for children included six, four, and five items for BE-weight, BE-appearance, and BE-attribution, respectively. Cronbach’s alphas for the BE-weight, BE-appearance, and BE-attribution subscales and the total scale were 0.85, 0.76, 0.69, and 0.85 respectively (Table 3). Table 3 provides the results for test-retest reliability in the separate subscales (r = 0.57–0.68, P < 0.01).

**Table 3:** Internal consistency (Cronbach’s alpha) and test-retest reliability (Spearman’s correlation coefficient) of the subscales for the Turkish version of the Body Esteem Scale for Adolescents and Adults for children.

Subscales	Spearman’s rhocorrelation coefficient	Cronbach’salpha
BE-Weight	0.68 (P < 0.01)	0.85
BE-Appearance	0.68 (P < 0.01)	0.76
BE-Attribution	0.57 (P < 0.01)	0.69
Overall scale		0.85

BE: Body esteem.

Table 4 shows the descriptive statistics for the three subscales and total scores by gender. The BE-appearance scores were statistically different between the genders (P = 0.001). The BE-appearance median score for girls [18.0 (0–24)] was higher than that for boys [16.0 (0–24)]. Similarly, the total scores were statically different for the genders (P = 0.04). The BE-total median score for girls [41.0 (0–60)] was higher than that for boys [39.0 (0–60)].

**Table 4:** Descriptive statistics and comparisons of BE-weight, BEappearance, and BE-attribution, and total score for the Turkish version of the Body Esteem Scale for Adolescents and Adults by gender (boys: n = 779, girls: n = 869).

Subscales	Sex	Median(min–max)	Mean (SD)	p
BE-Weight	Boys	15.0 (0–20)	13.6 (5.7)	0.342
	Girls	14.0 (0–20)	13.3 (5.8)	
	Total	14.0 (0–20)	13.4 (5.8)	
BE-Appearance	Boys	16.0 (0–24)	15.4 (6.4)	0.001
	Girls	18.0 (0–24)	16.4 (6.2)	
	Total	17.0 (0–24)	16.0 (6.3)	
BE-Attribution	Boys	10.0 (0–16)	10.0 (4.0)	0.228
	Girls	10.0 (0–16)	10.2 (3.7)	
	Total	10.0 (0–16)	10.1 (3.8)	
BE-Total	Boys	39.0 (0–60)	39.0 (11.5)	0.040
	Girls	41.0 (0–60)	40.0 (12.3)	
	Total	40.0 (0–60)	39.5 (11.9)	

BE: Body esteem.

## 4. Discussion

To our knowledge, this was the first study to examine the validity and reliability of the Turkish version of the BESAA for children. We confirmed the validity of the BESAA with three dimensions, consistent with the original scale of Mendelson et al. [1], but with a reduced number of items based on inappropriate or inadequate factor loadings. The final scale in our study demonstrated satisfactory goodness of fit across multiple indices and acceptable to good internal consistency and test-retest reliability, suggesting its applicability for use in the future research related to children.

The reduced number of items in our final model suggests the presence of cultural variations in commonly used scales and thus the importance of investigating the validity and reliability of measures for new populations. Although the scale was professionally edited by Turkish linguists, and a pilot study of the scale was conducted a priori, the children seemed to perceive some items in the BE-weight and BE-appearance subscales differently. Possibly, the concepts of some items in the BE-weight and BE-appearance subscales were not clear-cut or clearly distinct from each other in the Turkish language. Nonetheless, the reduced model showed good fit, and thus could be considered as a more parsimonious measurement.

We assessed two types of reliability: internal consistency and test-retest reliability. Internal consistency is generally considered acceptable if Cronbach’s alpha is greater than 0.6 [18–20]. In addition, Spearman’s rho correlation coefficient can be interpreted as moderate if it is between 0.40 and 0.69 and strong if it is between 0.70 and 0.89 [21]. Our results met all these criteria, with internal consistency values ranging from 0.68 to 0.85 and Spearman’s r ranging from 0.57 to 0.68. 

Our results corroborate the findings for Italian children by Confalonieri et al. [22], who through a factor analysis confirmed the three subscales with reduced items [BE-appearance (six items), BE-weight (four items), and BE-attribution (four items)]. 

However, in contrast to our findings, the 14-item Italian version of the BE-appearance and BE-weight scores for girls were lower than those for boys, suggesting cultural differences in BE between children in different countries. The study also showed that the Italian of the BESAA positively correlated with the Italian version of Rosenberg’s Self-Esteem Scale [22,23] and the Body Image Satisfaction Questionnaire [24]. 

Another study in Sweden also examined the importance of BE among children. Erling and Hwang [25] found a negative relationship between BE and BMI, and a negative relationship between BE and dieting among Swedish children aged 10 years. According to the study, BE can be affected by weight. Girls who were overweight had lower BE scores in all subscales than girls with normal weight. Like their Italian counterparts, Swedish girls had significantly lower BE scores than Swedish boys. Although beyond the scope of the current study, we plan to examine the relationship between BE and adiposity among children in the future.

According to the results of the validity and reliability analyses, the BESAA used in this study was confirmed as a useful and practical instrument to evaluate BE among children. Our results indicate that the BESAA Scale can be used to measure BE in terms of appearance, weight, and attribution in children, facilitating further research using this scale in the future. There is a great need to incorporate psychosocial components in the design of weight-related interventions in Turkey [26]. Our study contributes to the science needed to build the evidence base for prevention and treatment programs in Turkey.

## Acknowledgments

We thank Hacettepe University Institute of Public Health, Ankara, Turkey, and the University of Nebraska Medical Center, USA, for their research support. The project (TUA-2015-5521) was supported by funding from the Scientific Research Projects Coordination Unit of Hacettepe University and by the University of Nebraska Office of the President as a part of their commitment to global collaboration.
